# Extending laying hens’ productive lifespan by molting: a pilot on-farm study and test of assessment methods with focus on functional traits and keel bone health

**DOI:** 10.1016/j.psj.2025.106323

**Published:** 2025-12-20

**Authors:** Julia Gickel, Frieder Broszat, Louisa Salzmann, Nicole Kemper, Lisa Jung

**Affiliations:** a Institute for Innovative and Sustainable Poultry Farming (WING), University of Veterinary Medicine Hannover, Foundation, 30173 Hannover, Germany; b Department of Animal Breeding, University of Kassel, 37213 Witzenhausen, Germany; c Institute for Animal Hygiene, Animal Welfare and Farm Animal Behaviour (ITTN), University of Veterinary Medicine Hannover, Foundation, 30173 Hannover, Germany

**Keywords:** Molting, Laying hen production, Laying hen welfare, Keel bone damage, On-field assessment

## Abstract

This study investigated potential methods to test the effects of induced molting on performance, health, and welfare traits in two mobile-housed end-of-lay flocks on individually marked hens under practical conditions. Flock 1 consisted of 124 dual-purpose Coffee & Cream hens managed organically with continued free-range access during molting. Flock 2 included 162 Lohmann Brown hens under conventional conditions without free-range access during molting. Molting protocols differed markedly in duration and severity. Molting was induced by restricting feed and light. Health, performance, and keel bone traits were assessed before, during, and after molting using individual marking, radiography, egg and feed analyses, and endoparasite diagnostics. Laying performance declined during molting in both flocks and partially recovered afterwards (flock 1: from 54% to 30% to 39%; flock 2: from 89% to 0% to 65%). Egg quality parameters improved post-molt in both flocks, suggesting recovery of reproductive tract function. Body weight decreased throughout molting and increased significantly thereafter in both flocks, indicating physiological recovery. However, keel bone fracture prevalence increased significantly from before to after molting in both flocks, despite stable or improved bone mineral density (BMD). Comb injuries increased significantly in flock 2 post-molt, likely due to abrupt feed withdrawal and lack of free-range access. Conversely, flock 1, managed under a gradual molting protocol, exhibited calmer behavior and general improvement in integument scores. Feed analyses revealed presumably suboptimal calcium-to-phosphorus (Ca:P) ratios in both flocks, which may have limited skeletal recovery. Because molting occurred in different seasons, photoperiod and climatic conditions may have moderated cohort responses and should be interpreted as contextual field effects rather than causal treatment differences. This study highlights that molting outcomes are associated with flock condition and the applied molting protocol. Furthermore, the methods of individual marking and X-raying of hens proved to be applicable and informative, provided that suitable leg rings are used and consistent X-ray settings are maintained. However, a sufficiently large sample size is required to compensate for potential losses, and it should be taken into account that hens are particularly sensitive during the molting period.

## Introduction

Egg production constitutes a significant sector in food security and agricultural economics (Mottet and Tempio, 2017). Approximately 7.9 billion laying hens produced 86.7 tonnes of shell eggs in 2020 worldwide (Compassion in Food Business, 2023). By 2023 this increased to 91 million tonnes of eggs (FAO, 2025), to meet increasing consumer demand driven by population growth and dietary changes. Optimizing the productivity of laying hens is vital for ecological and economic sustainability, but animal welfare is also of growing consumer interest (Rahmani et al., 2019; Gautron et al., 2021). Breeding companies have progressively extended laying hen productive lifespan from average production ending around 70–75 weeks in past decades, to current targets of 90–100 weeks and 500 eggs per hen per cycle (Bain et al., 2016; Lohmann Breeders, 2021). A prolonged laying period may offer both sustainability and economic benefits, depending on factors such as laying performance and egg quality. Studies have shown that modern laying hens are capable of maintaining stable performance over extended cycles, with average laying rates of 74–76% and peak values of 86–89% reported between 68 and 97 weeks of age, while egg quality traits remain commercially acceptable until around 80 weeks (Gwendolyn, 2018; Traore and Doyon, 2023). In general, egg quality traits such as eggshell thickness and strength decline with increasing hen age due to changes in shell ultrastructure and matrix protein composition (Roberts, 2004; Ahmed et al., 2005). Animal health remains a critical consideration when extending the laying cycle, as prolonged production periods can increase the risk of metabolic disorders, skeletal problems, and infectious diseases in hens (Bain et al., 2016; Korver, 2023). For instance, longer laying cycles may exacerbate osteoporosis and keel bone damage due to extended calcium mobilization for eggshell formation (Richards et al., 2011). An alternative to a prolonged productive lifespan, especially for smaller and organic systems, could be a second or even third laying cycle reached by a laying break through induced molting. Molting is a natural, cyclical process in birds during which reproductive activity ceases temporarily, accompanied by feather loss and physiological changes. Commercially, induced molting was used to rejuvenate flocks, extend laying cycles, and improve post-molt egg quality (Berry, 2003; Wang et al., 2024). Economically, molting offers a strategy to reduce pullet replacement costs and improve overall flock profitability. Post-molt hens often demonstrate enhanced egg production and improved shell quality, though results depend on molt method, age, and breed (Bar et al., 2001; Ahmed et al., 2003; Berry, 2003). Historically, molting was induced via feed withdrawal, causing a forced cessation of lay, but ethical concerns and welfare issues associated with hunger and stress led to alternative strategies involving non-fasting diets rich in fiber (Koelkebeck and Anderson, 2007; Mishra et al., 2022). While molting can improve post-molt egg production and quality, the associated physiological stress and potential welfare implications raise significant concerns. However, Webster (2000) concluded that, depending on the method used, induced molting does not necessarily harm hens and may even enhance their survivability. The effectiveness of alternative molting methods varies, and there remains an ongoing debate about their relative benefits and drawbacks. Health indicators such as plumage condition, footpad status, and keel bone integrity are frequently monitored to assess hens’ wellbeing (Welfare Quality Network, 2019). In particular, keel bone damage affects a large proportion of commercial laying hens and is associated with pain, reduced mobility, and compromised productivity (Casey-Trott and Widowski, 2016; Rentsch et al., 2019). Studies indicate that bone density loss during molting can increase fracture risk, compromising both welfare and productivity (Mazzuco and Hester, 2005). However, based on previous findings of increased calcium absorption in the duodenum after molt (Al-Batshan et al., 1994) and of elevated levels of the hormonal form of vitamin D together with enhanced tissue responsiveness (Berry, 2003), it can be hypothesized that molting may positively influence keel bone health.

The existing literature highlights a gap in the multifaceted and longitudinal studies investigating the welfare, health, and productivity impacts of molting under commercial conditions. Specifically, the interplay between keel bone health, plumage condition, footpad health, and egg quality throughout the molting cycle requires further elucidation. Moreover, welfare implications linked to different molting methods remain insufficiently characterized.

This study examined the effects of induced molting on performance, health, and welfare traits in two mobile-housed end-of-lay flocks under practical conditions. Beyond answering these scientific questions, the research also focused on evaluating practical procedures, including hen marking, handling and catching hens during the sensitive molting period, radiography, and inducing molt within commercial practice settings. To our knowledge, this is the first true commercial on-farm pilot study combining individual hen marking with repeated keel-bone radiography during/after molting.

## Animals, materials and methods

### Ethical statement

All procedures involving animals were conducted in accordance with national and institutional guidelines for the care and use of animals and were approved by the competent authority. The study was carried out on farms with the owners' consent and under commercial conditions. Ethical approval was obtained from the relevant authority: Regierungspräsidium Kassel, approval number: RPKS-23-19 c 1612/22-2024/1.

### Animals, housing and molting

The longitudinal on-farm field study was conducted between July 2024 and January 2025 and included two commercial end-of-lay flocks of individually marked hens, with individual hens as the statistical unit. The first flock (flock 1) consisted of the dual-purpose genotype Coffee & Cream (Ökologische Tierzucht gGmbH, Augsburg, Germany), which is a crossbreed of New Hampshire × Bresse (Coffee) and White Rock × Bresse (Cream). This flock was housed in a Kerkstroer mobile coop (Kerkstroer Mobilstall GmbH & Co. KG, Rietberg, Germany) and managed under organic farming regulations. It comprised 124 hens and 2 roosters. The second flock (flock 2) consisted of Lohmann Brown hens (Lohmann Breeders GmbH, Cuxhaven, Germany), kept in a Farmermobil mobile coop (farmermobil GmbH, Laer, Germany) under conventional management conditions, with a total of 162 hens. Both flocks had access to outdoor areas before and after the molting period. Additionally, the Coffee & Cream flock maintained outdoor access during molting. In flock 2, hens were dewormed with Fluboral (active substance: Flubendazole; Dechra Regulatory B.V., Bladel, Netherlands), for 5 days, directly before molting was induced.

In flock 1, molting was induced over a four-week period by gradually increasing the proportion of oats in the feed from 30% to 66%, while keeping the light in the mobile coop completely off throughout. In flock 2, molting was induced in a single day by simultaneously turning off the light, closing outdoor access, and replacing the regular feed with crushed summer barley. To terminate the molt in flock 1, the light duration was gradually increased back to 16 hours, and laying mash was reintroduced by mixing it with oats over a period of several days until 100% laying mash was reached. In flock 2, molting was ended by providing a 50/50 mixture of crushed summer barley and laying mash followed by a complete switch to 100% laying mash over a ten-day period. Simultaneously, lights were turned on in the mobile coop and outdoor access was restored.

Both molting approaches applied in this study are consistent with previously described non-fasting or diet-based molt induction strategies. Gradual dietary dilution using low-nutrient grains (e.g., oats, barley, wheat) in combination with reduced photoperiod has been shown to effectively induce molt while maintaining acceptable physiological stress levels in hens (Biggs et al., 2004; Glatz and Tilbrook, 2021). Similarly, single-step dietary switches to nutrient-diluted grains, together with abrupt light reduction, have been reported as valid non-fasting methods that reliably suppress lay and trigger follicular regression (Zhang et al., 2022; Lei et al., 2023). Therefore, the procedures used in both flocks align with established, peer-reviewed molting protocols and reflect recognized refinements of non-fasting molt induction techniques.

### Assessments

**Fig. 1:**
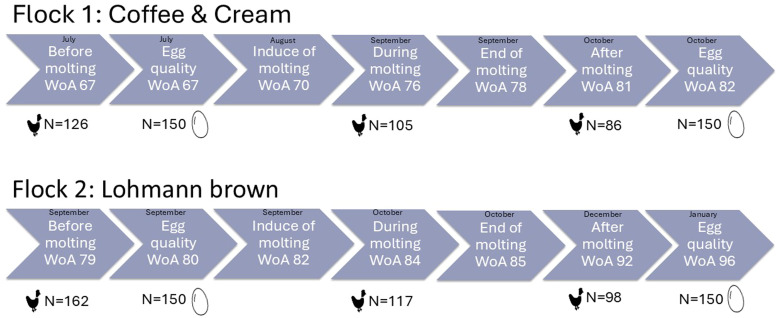
Study design showing flocks, week of age (WoA), sample size and month of the health assessment before, during and after molting and time point and sample size for egg quality analyses.

To evaluate differences in health indicators and body weight before, during, and after molting, three assessments were conducted in each flock at the individual bird level. The assessment time points, during and after molting were determined based on laying performance. In flock 2, it was straightforward to wait until laying performance declined to 0% before conducting the “during molting” assessment. In flock 1, however, laying performance was already low and decreased only gradually. Therefore, the second assessment was conducted at 30% laying performance, as no further decline was observed. The post-molting assessment was scheduled within the project’s time frame and took place once farmers reported no further increase in laying performance. Each hen was individually marked with a numbered leg ring during the first assessment. In addition, air quality in the coop, feed quality, endoparasite burden, and egg quality were examined. Fig. 1 displays the hens’ week of age (WoA), sample sizes, and the corresponding assessment months.

Health indicators (including injuries/feather loss at comb, back, cloaca, and footpads) were monitored at every assessment. All of their scores and definitions are listed in Table 1. All health assessments were consistently performed by the same well-trained person who conducted various inter- and intra-observer-reliability tests for different studies (see, e.g. Gieseke et al., 2022; Jung et al., 2024). Scores were entered on an outdoor tablet using the Data Collection Tool app from the Google Play Store (Volk, 2025). The assessment protocol is comparable with the KTBL assessment protocol for Poultry (Knierim et al., 2020).

**Table 1:** Definition of health indicators and corresponding scores.

**Indicator**	**Score 0**	**Score 1**	**Score 2**	**Score 3**
**Laying status**	Not laying: ≤2 fingers between keel and pubic bones; small, tight cloaca	Laying: >2 fingers between keel and pubic bones; large, elastic cloaca		
**Molting status**	Full plumage; no feather loss on head, neck or sides; no new feather growth (except due to feather pecking)	Featherless areas on head, neck and/or sides; new feather growth	Small areas with new feather growth; not typical for feather pecking	
**Comb**	No injuries	1 to 3 small injuries	>3 small injuries or ≥1 injury >0.5 cm	
**Eye/Noistrils**	Clear eyes, no nasal discharge	Swollen, cloudy or purulent eyes/nasal discharge		
**Beak**	Intact, rounded beak	Overgrown, pointed or cracked beak		
**Neck** f**eather**	No featherless areas; ≤1 missing feather	Featherless area >1 to <5 cm; pinfeathers may be visible	Featherless area ≥5 cm; pinfeathers may be visible	
**Back feather**	No featherless areas; ≤1 missing feather	Featherless area > 1 to <5 cm; pinfeathers may be visible	Featherless area ≥5 cm; pinfeathers may be visible	
**Back skin**	No injuries	≤2 injuries ≤1 cm (fresh or crusted)	≥3 injuries ≤1 cm or ≥1 injury >1 cm (fresh or crusted)	
**Cloaca/belly feather**	No featherless areas; ≤1 missing feather	Featherless area > 1 to <5 cm; pinfeathers may be visible	Featherless area ≥5 cm; pinfeathers may be visible	
**Cloaca/belly skin**	No injuries	≤2 injuries ≤1 cm (fresh or crusted)	≥3 injuries ≤1 cm or ≥1 injury >1 cm (fresh or crusted)	
**Cloaca/belly soiling**	Clean feathers	≤3 soiled feathers	>3 soiled feathers	
**Toes**	Intact toes, no amputation or visible injuries	Lesion or amputation on upper/lower side of a toe		
**Footpad**	Intact	Hyperkeratosis	Ulceration	Swelling/Abscess

Body weight was measured in grams (g), by attaching a bucket to a BAT VEIT Electronics scale (VEIT Electronics, s.r.o., Moravany, Czech Republic). Hens were gently placed into the bucket, and weights were recorded on the tablet.

Air quality was monitored throughout the entire project period by installing a data logger (Log32 TH, TFA Dostmann GmbH + Co. KG, Wertheim, Germany) in each mobile coop unit, positioned at the drinker line between two cups. Data were downloaded from the logger and subsequently stored and analyzed using the statistical software JMP, where plots of the measured variables were generated. For feed quality analysis, 500 g of feed were sampled directly from the feed container on the day of assessment and sent to LKS – Landwirtschaftliche Kommunikations- und Servicegesellschaft mbH, Feed and Soil Laboratory, Niederwiesa, Germany, for analysis of nutrient composition. In cases where multiple containers were used, composite samples were created by taking equal subsamples from each container.

To assess endoparasite load, 50 fresh fecal droppings were randomly collected both indoors and outdoors during each assessment. Samples were pooled and submitted to LUFA Nord-West, Institute for Animal Health, Oldenburg, Germany, for endoparasite analysis using the flotation method. Endoparasites were determined at the species level considering roundworm eggs (*Ascaridia galli*), coccidia oocysts (*Eimeria* spp.), hairworm eggs (*Capillaria* spp*.*), and tapeworm eggs (*Davainea proglottina, Raillietina* spp*.*). Here, “spp.” denotes that multiple species within the genus may occur.

Egg quality was assessed from each flock on 150 eggs before and after molting, employing the analysis techniques provided by Bröring Technology GmbH, Lohne, Germany. Unsorted eggs were randomly collected by the farmers on three consecutive days. Egg weight was measured in g, using the Kern 440-43 N laboratory scale (Kern & Sohn GmbH, Germany) with a maximum capacity of 400 g. Eggshell strength was assessed in N using the Fast-Egg-Shell-Tester (FEST, Bröring Technology GmbH, Germany). Eggshell thickness was measured in µm with the Eggshell Thickness Tester (Ref. 1505, Bröring Technology GmbH, Germany). Yolk color was assessed using an RGB-based yolk color measurement device (Bröring Technology GmbH, Germany); color values are expressed in L*a*b* coordinates and Roche units (1–15). Albumen height was measured 1 cm from the yolk edge on a flat glass plate using the EggQuality 3.0 system (Bröring Technology GmbH, Germany); height is reported in millimeters (mm). Haugh units were calculated using the formula: HU=100×log10 (*h* − 1.7w^0.37+7.6), where h is albumen height in millimeters (mm) and w is egg weight in grams (g).

### Radiograph evaluation of the keel bone

Keel bones were radiographed in the free-range area using a mobile X-ray unit (Amadeo P90 20VB; Oehm und Rehbein GmbH, Rostock, Germany). A custom-built suspension frame was used to position the hens during imaging, as illustrated in supplementary material (S1). For the evaluation of bone density, a 17-step aluminum step wedge was attached to the detector plate as described in Eusemann et al. (2020) (see S2).

The radiographs were saved at the individual level and assigned to the three respective time points (before, during, after molting). For the analysis of keel bone traits, the software ImageJ (version 1.54 g, National Institutes of Health, Ort, USA) was used. All radiographs were evaluated by the same well-trained examiner who was trained according to Rufener et al. (2018) for fractures and Jung et al. (2022) for deviations. The following keel traits were assessed: fractures (0 = no fracture, 1 = one fracture, 2 = multiple fractures, 3 = single fractures not identifiable due to total damage) that were further differentiated into two types: (i) fractures showing a radiolucent line or dislocation without visible callus formation, and (ii) fractures with callus formation but no visible fracture gap (characterized by irregular bone margins, whereby a keel could also suffer from both types), fracture location (A, B, C), keel length, bone density (mm Al), and area of deviation (cm²). For further analysis, both types (i and ii) were classified as a fracture and added to one single value per hen. Bone density was assessed by converting grayscale values from radiographs into aluminum equivalents (mm AlEq) using the macro “Calibrate Grey Values”, which generated a standard curve based on an aluminum step wedge. The macro “Square Tool” was then used to draw a line from the cranial to the caudal tip of the keel bone (or the caudal end of the ossified part), automatically placing a square at its midpoint where the mm AIEq was measured. For deviations, the entire keel bone area was manually outlined. A straight line was drawn along the presumed anatomically correct axis of the keel bone to serve as a reference. The area of deviation was subsequently determined as the space between this ideal axis and the actual contour of the bone, allowing for quantification of the deformation in square centimeters. The classification of the deformations can be found in the supplementary material (S3 A-D shows A: a fracture, B: a callus formation, C: a deviation and D: the locations).

The aluminum step wedge served as the reference for bone density measurements as well as for distance calibration in centimeters.

### Statistical analyses

Differences in hen weight, bone density, and deviations were assessed using ANOVA following a post-hoc Bonferroni test, differences in egg quality (egg weight, eggshell thickness, and breaking strength) using a t-test. The differences in all health indicators were assessed using a cumulative link model with the time point as a predictor variable.

Region-specific fracture scores (A–C) were evaluated first. A total keel fracture score per hen was then derived by summarizing across regions, such that the presence of fractures at ≥2 sites was classified as ‘multiple fractures’ (score 2) even when individual sites were scored as 1; score 0 and 1 indicated no or a single fracture across A–C, respectively, and score 3 indicated total damage preventing identification of single fractures. Correlations of bone density to fractures were assessed using Spearman's rank correlation rho. All calculations were performed using the statistical program R (v4.3.0) (R Core Team, 2024) including the packages “emmeans”, “ggplot2”, “gridExtra”, “lmerTest”, “readxl” and “tidyverse”. A p-value of <0.05 was considered as statistically significant.

## Results

### Performance parameters

#### Laying performance

In flock 1, laying performance was recorded at 54.0% before molting (67 weeks of age), decreased to 30.0% during molting (76 weeks of age; five weeks after the onset of molting), and increased to 39.0% after molting (82 weeks of age; three weeks after the end of molting). In flock 2, laying performance was 89.0% before molting (79 weeks of age), dropped to 0% during molting (84 weeks of age; two weeks after the onset of molting), and increased again to 65.0% after molting (92 weeks of age; seven weeks after the end of molting).

#### Weight

**Fig. 2:**
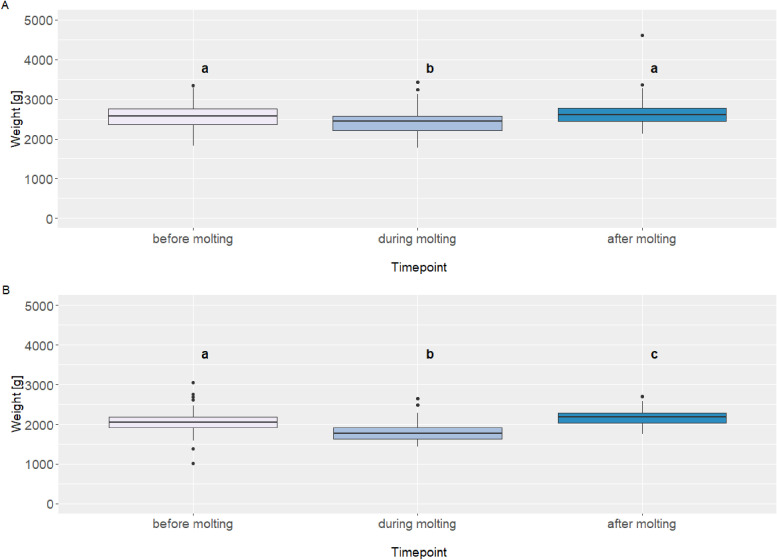
Weight (g) of flock 1 and flock 2 during the observation period.

In flock 1, the mean body weight was 2,585 ± 25.3 g before molting, which decreased to 2,421 ± 30.6 g during molting, and increased to 2,651 ± 37.7 g after molting (see Fig. 2A). Pairwise comparisons showed that body weight during molting was significantly lower than both before molting, t(84) = 3.72, *p* = 0.001, and after molting, t(84) = −4.13, *p* < 0.001. However, no significant difference was found between before and after molting, t(84) = −0.85, *p* = 0.40. In flock 2, the mean body weight was 2,060 ± 18.8 g before molting, dropped to 1,785 ± 19.2 g during molting, and increased to 2,176 ± 19.5 g after molting (see Fig. 2B). Pairwise comparisons indicated that all three timepoints differed significantly: weight during molting was lower than before molting, t(84) = 12.2, *p* < 0.001, and after molting, t(84) = −17.4, *p* < 0.001, while weight after molting was also significantly higher than before molting, t(84) = −4.52, *p* < 0.001.

#### Egg quality

In flock 1, the mean egg weight was 67.5 ± 5.5 g before molting and significantly increased to 68.9 ± 5.9 g after molting. In flock 2, the mean egg weight increased significantly from 64.2 ± 5.4 g before molting to 69.2 ± 5.9 g after molting. The eggshell thickness in flock 1 remained unchanged, with values of 0.36 ± 0.03 mm before and 0.36 ± 0.04 mm after molting (not significant). In contrast, flock 2 showed a significant increase in eggshell thickness from 0.35 ± 0.04 mm before molting to 0.38 ± 0.05 mm after molting. Regarding shell breaking strength, no significant difference was observed in flock 1 (32.7 ± 9.4 N before vs. 31.4 ± 8.6 N after molting). However, in flock 2, breaking strength significantly increased from 31.8 ± 9.6 N before molting to 42.3 ± 11.7 N after molting.

### Health indicators

#### Comb injuries

**Fig. 3:**
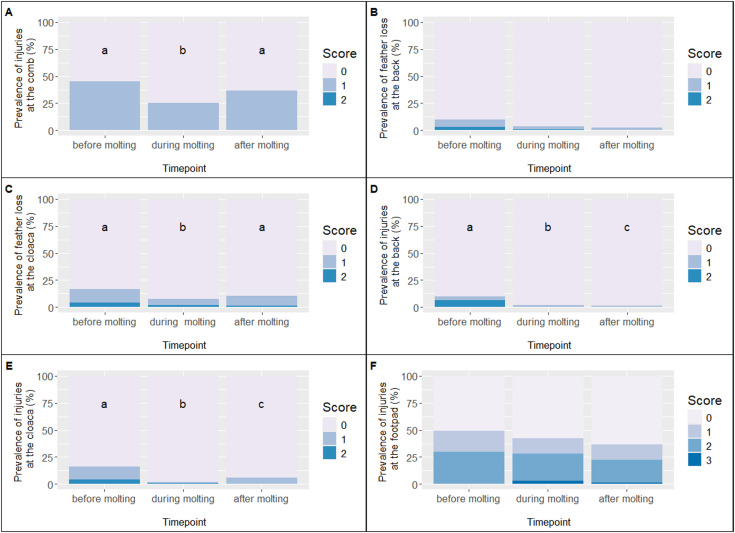
Prevalence of scores for the health indicators in flock 1.

**Fig. 4:**
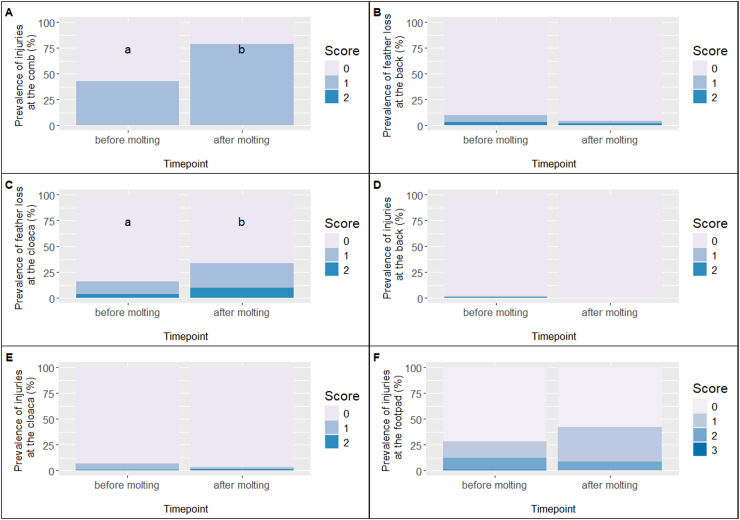
Prevalence of scores for the health indicators in flock 2.

In flock 1, the prevalence of comb injuries (score 1) was 45.2% before molting, which dropped to 25.0% during molting, and increased again to 36.5% after molting (see Fig. 3A). An ordinal logistic regression revealed a significant reduction in comb injuries during molting compared with before molting, β = −0.90, SE = 0.29, *z* = −3.12, *p* = 0.002, while no significant difference was observed between during and after molting, β = −0.36, SE = 0.29, *z* = −1.25, *p* = 0.21. In flock 2, the prevalence of comb injuries increased markedly from 42.6% before molting to 78.5% after molting (see Fig. 4A). The ordinal logistic regression indicated that the risk of comb injuries was significantly higher after molting compared to before, β = 1.59, SE = 0.30, *z* = 5.34, *p* < 0.001. All other animals in both flocks were consistently classified as score 0 (no comb injury).

#### Back feather loss

In flock 1, the prevalence of feather loss on backs was 6.5% (score 1) and 3.2% (score 2) before molting, 2.9% (score 1) and 1.0% (score 2) during molting, and 2.4% (score 1) after molting (see Fig. 3B). Ordinal logistic regression indicated no statistically significant differences between the time points. The contrast “before vs. during molting” showed a trend-level reduction, β = −0.99, SE = 0.59, *z* = −1.67, *p* = 0.095, and “before vs. after molting” was similarly non-significant, β = −1.50, SE = 0.78, *z* = −1.94, *p* = 0.053. In flock 2, feather loss was 6.8% (score 1) and 3.1% (score 2) before molting, and 2.2% (score 1) and 2.2% (score 2) after molting (see Fig. 4B). Ordinal logistic regression confirmed that differences between the two time points were not statistically significant, β = −0.88, SE = 0.57, *z* = −1.53, *p* = 0.126. The majority of animals in both flocks were consistently classified as score 0 (no feather loss at the back).

#### Cloaca feather loss

In flock 1, the prevalence of feather loss at the cloaca was 12.9% (score 1) and 4.0% (score 2) before molting, 5.8% (score 1) and 1.9% (score 2) during molting, and 9.4% (score 1) and 1.2% (score 2) after molting (see Fig. 3C). The statistical model confirmed that the prevalence during molting differed significantly from the other time points (Bonitur2: *p* = 0.041). In flock 2, the prevalence was 12.3% (score 1) and 3.7% (score 2) before molting, and 23.7% (score 1) and 9.7% (score 2) after molting (see Fig. 4C). Here, the model also indicated a significant increase in feather loss before compared to after molting (Bonitur3: *p* = 0.002). All other hens in both flocks were classified with score 0.

#### Back injuries

In flock 1, the prevalence of injuries on the back was 3.2% (score 1) and 6.5% (score 2) before molting, 1.9% (score 1) during molting, and 1.2% (score 1) after molting (see Fig. 3D). The ordinal regression model confirmed that all time points differed significantly from each other (Bonitur2: *p* = 0.026; Bonitur3: *p* = 0.035). In flock 2, the prevalence after molting was 1.2% (score 1) and 0.6% (score 2) (see Fig. 4D), and no significant differences between time points were detected. All other hens in both flocks were classified with score 0 for injuries on the back.

#### Cloaca injuries

In flock 1, the prevalence of injuries at the cloaca was 12.1% (score 1) and 4.0% (score 2) before molting, 1.0% (score 1) and 1.0% (score 2) during molting, and 5.9% (score 1) after molting (see Fig. 3E). The ordinal regression model confirmed that all time points differed significantly from each other (Bonitur2: *p* = 0.003; Bonitur3: *p* = 0.029). In flock 2, the prevalence was 6.2% (score 1) and 0.6% (score 2) before molting, and 2.2% (score 1) and 1.1% (score 2) after molting (see Fig. 4E), with no significant differences between the two time points. All remaining animals in both flocks were classified with score 0 for injuries at the cloaca.

#### Footpad injuries

In flock 1, the prevalence of footpad injuries was 19.4% (score 1) and 29.8% (score 2) before molting; 14.4% (score 1), 25.0% (score 2), and 2.9% (score 3) during molting; and 14.1% (score 1), 21.2% (score 2), and 1.2% (score 3) after molting (see Fig. 3F). The ordinal regression model did not reveal significant differences between all time points (Bonitur2: *p* = 0.478; Bonitur3: *p* = 0.097). In flock 2, the prevalence was 15.4% (score 1), 11.7% (score 2), and 0.6% (score 3) before molting, and 33.3% (score 1) and 8.6% (score 2) after molting. Again, no significant differences were observed between time the points (Bonitur3: *p* = 0.073). The remaining birds in both flocks were classified as score 0, indicating no visible footpad injuries (see Fig. 4F).

#### Mortality

Based on farmers information the mortality rate was 7.8% in flock 1 and 7.2% in flock 2 during the project time.

### Keel bone health

#### Fractures

**Fig. 5:**
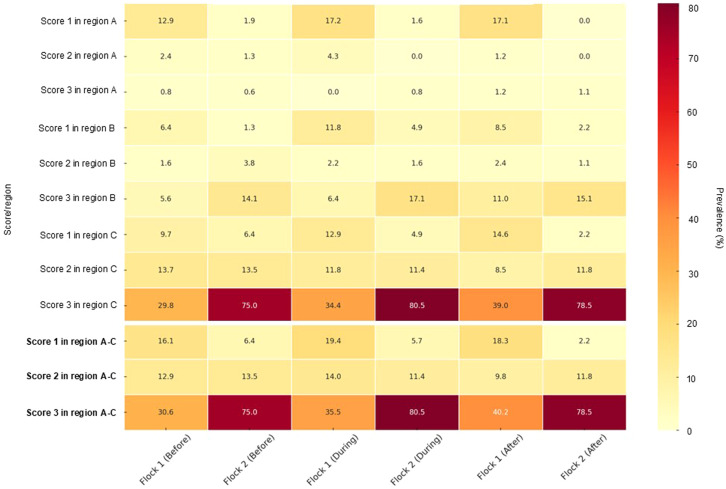
Prevalence of fractures in flock 1 and flock 2.

In both flocks, the fracture rates increased from before to after molting. The prevalences are summarized in Table 2 and illustrated in a heatmap in Fig. 5.

**Table 2:** Fracture prevalences of the keel bone of a dual-purpose cross breed (Coffee & Cream, Flock 1) and a Lohmann Brown flock (flock 2) before, during and after molting at three different locations (A, B, C) with score 0 = no fracture, 1 = one fracture, 2 = multiple fractures, 3 = single fractures not identifiable due to total damage.

Flock	Region	Score	Before molting (%)	During molting (%)	After molting (%)
1	A	1	12.9	17.2	17.1
		2	2.42	4.3	1.22
		3	0.81	0	1.22
	B	1	6.45	11.8	8.54
		2	1.61	2.15	2.44
		3	5.65	6.45	11.0
	C	1	9.68	12.9	14.6
		2	13.7	11.8	8.54
		3	29.8	34.4	39.0
	Total	1	16.1	19.4	18.3
		2	12.9	14.0	9.76
		3	30.6	35.5	40.2
2	A	1	1.92	1.63	0
		2	1.28	0	0
		3	0.64	0.81	1.08
	B	1	1.28	4.88	2.15
		2	3.85	1.63	1.08
		3	14.1	17.1	15.1
	C	1	6.41	4.88	2.15
		2	13.5	11.4	11.8
		3	75.0	80.5	78.5
	Total	1	6.41	5.69	2.15
		2	13.5	11.4	11.8
		3	75.0	80.5	78.5

#### Bone density

**Fig. 6:**
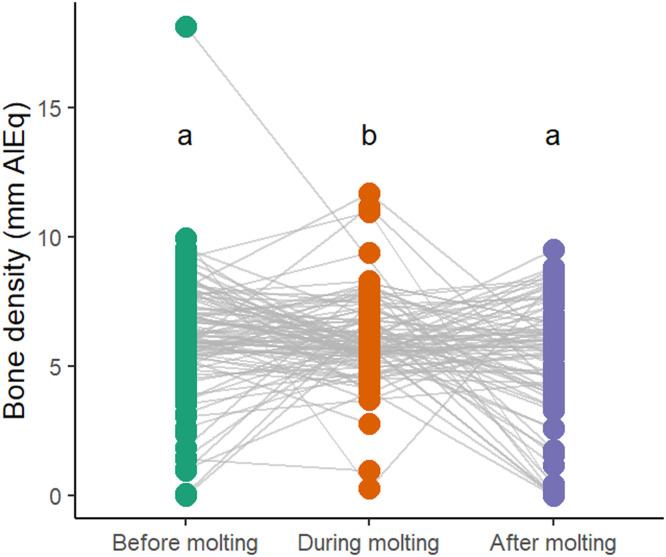
Keel bone density (mm AlEq) of flock 1 during the observation period.

**Fig. 7:**
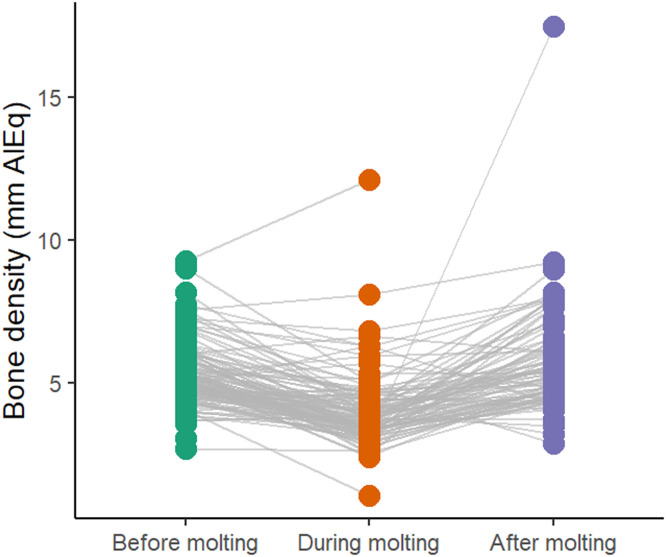
Keel bone density (mm AlEq) of flock 2 during the observation period.

In flock 1, the mean bone density was 6.1 ± 2.2 mm AlEq before molting, 6.1 ± 1.7 mm AlEq during molting, and 5.2 ± 2.5 mm AlEq after molting. The mixed model revealed the time point to be a significant effect (F (2,299) = 4.61, *p* = 0.011). Post-hoc comparisons indicated that bone density was significantly lower after molting compared to both before (*p* = 0.016) and during molting (*p* = 0.029). No difference was found between the assessments before and during molting (*p* = 0.997; see Fig. 6). In flock 2, the mean bone density was 5.2 ± 1.1 mm AlEq before molting, 4.0 ± 1.2 mm AlEq during molting, and 5.7 ± 1.9 mm AlEq after molting. The time points effect was strong (F (2,245) = 68.15, *p* < 0.001), and post-hoc tests showed that bone density during molting was significantly lower than both before (*p* < 0.001) and after molting (*p* < 0.001). In addition, bone density after molting was higher than before molting (*p* = 0.014; see Fig. 7).

#### Deviations

In flock 1, the mean area of deviations was 0.50 ± 0.62 cm^2^ before molting, 0.60 ± 0.51 cm^2^ during molting, and 0.66 ± 0.60 cm^2^ after molting, without significant differences. In flock 2, the mean area of deviations was 0.29 ± 0.38 cm^2^ before molting, 0.29 ± 0.35 cm^2^ during molting, and 0.34 ± 0.47 cm^2^ after molting, also without significant differences.

#### Correlation of bone density and deviations to fractures

In both flocks, no significant correlations were found between bone density and the occurrence of fractures (neither total score nor scores per anatomical region). Accordingly, no relationship between fracture scores and bone density was confirmed. For the area of deviations, however, significant positive correlations were found. In flock 1, correlations were detected with the total fracture score (rₛ = 0.20, *p* < 0.01), the score of fractures in region B (rₛ = 0.14, *p* = 0.01), and in region C (rₛ = 0.15, *p* < 0.01). In flock 2, significant correlations were found for region B (rₛ = 0.12, *p* = 0.02) and region C (rₛ = 0.12, *p* = 0.02). All correlations indicated that larger deviation areas were associated with a higher occurrence of fractures.

### Further conditions

#### Air quality

During the observation of flock 1, the temperature varied between 5.0 °C and 35.3 °C. The mean value was 18.9 °C. In flock 2, the temperature varied between 0.8 °C and 28.7 °C with a mean of 13.6 °C. The mean dew point was higher for flock 1 (14.7 °C compared to 10.3 °C) and ranged from 3.2 °C to 27.2 °C (flock 1) compared to −1.1 °C to 20.6 °C for flock 2. The mean relative humidity was 78.3% (range: 37.3% to 100%) for flock 1 and 81.2% (range: 38.5% to 100%) for flock 2. The comparison of both flocks indicates a higher variation of measured values in flock 1 (see S4).

#### Feed analysis

The feed sample of flock 1 before molting (*n* = 1) contained a higher amount of calcium and a higher calcium-phosphorus-ratio than after molting (Table 3). For flock 2 the values appear comparable. Overall, crude protein was higher for flock 2 than for flock 1 and the calcium level was higher for flock 1. The levels of phosphorus and calcium-phosphorus-ratio showed no clear trend.

**Table 3:** Extract from the analytical ingredients per kg dry matter (DM) of feed samples from different time points (*n* = 1 per time point).

Ingredients (g per kg DM)	Flock 1			Flock 2^1^	
	Before molting	During molting	After molting	Before molting	After molting
Dry matter	916	897	897	893	887
Crude protein	139	138	173	192	185
Calcium	101.2	41.1	37.0	25.5	29.5
Phosphorus	4.8	12.6	8.5	4.8	5.4
Calcium-phosphorus-ratio (calculated)	21.1	3.3	4.4	5.3	5.5
Lysine	6.3	5.5	7.6	9.6	8.1
Methionine	2.6	2.1	3.2	3.5	2.9
Cysteine	2.3	2.6	3.4	2.8	6.1
Threonine	4.9	4.5	6.1	7.2	3.9
Valine	6.2	6.0	8.2	8.3	6.8
Histidine	3.2	2.8	4.2	4.4	3.7
Metabolizable energy (calculated; MJ per kg DM)	8.6	9.3	12.2	13.6	13.4

1 no feed sample could be obtained during molting in flock due to low filling quantity of the feed chain.

#### Endoparasite analysis

Table 4 provides an overview of the presence of various endoparasites detected in the fecal samples collected from the two flocks at three time points relative to the molting period. In general, lower endoparasite burdens were observed after molting compared to before.

**Table 4:** Quantity of endoparasite eggs and oocysts in fecal samples from flocks 1 and 2 before, during, and after the molting period.

Parasite Type	Flock1			Flock 2		
	Before molting	During molting	After molting	Before molting	During molting	After molting
Roundworm eggs (*Ascaridia galli*)	++	+	++	+	+	+
Coccidia oocysts (*Eimeria spp.*)	+	+	+	-	++	-
Hairworm eggs (*Capillaria spp.*)	+	+	-	-	-	-
Tapeworm eggs (*Davainea proglottina, Raillietina spp.*)	-	-	-	+	-	-

++ major evidence, + minor to moderate evidence, - no evidence.

## Discussion

The novelty of this pilot field study lies in implementing longitudinal individual-level radiography in a true commercial setting, where previous work was experimental or relied on flock-level sampling. This study examined both the practicability of methods such as individual marking, catching, and radiographing of hens, and the impact of induced molting on functional traits, with a special focus on keel bone health under commercial conditions. Two flocks with distinct characteristics were examined: Flock 1 was a dual-purpose breed with a heterogeneous population and free-range access during molt, while flock 2 was a commercial laying hen strain kept conventionally without free-range access during molt. Due to these inherent differences, direct genetic comparisons between the flocks are not possible. Accordingly, our primary inference is based on longitudinal within-flock, individual-hen pre- to post-molt changes; comparisons between flocks are interpreted descriptively as contextual field patterns. During the first assessment, some hens in flock 1 were already in molt, indicated by pale combs, partial feather loss on the head, neck and underwing regions, and changes in cloaca size and pubic bone spacing (assessment data presented in supplementary S1). Notably, some hens continued laying eggs during the molting period, while others remained in molt in the later assessment. Regarding laying performance, flock 1 showed a decline of approximately 15%-points three weeks post-molt compared to pre-molt levels. Flock 2 experienced a more pronounced decrease of about 24%-points. Seven weeks after molting, flock 2 reached a laying rate of 65%. This rate is lower compared to other post-molt laying rates reported in the literature. For example, Ramos et al. (1999) reported around 75%–85% between 8 and 12 weeks after molt. Alodan and Mashaly (1999) observed that induced molting increased laying rates from around 64% pre-molt to between 77% and 83% at 10 weeks post-molt. Biggs et al. (2003) documented that nonfeed removal methods for molting resulted in post-molt production levels of 66-77%. More recently, Tiwary et al. (2019) reported peak post-molt laying rates of about 88% achieved approximately 9 weeks after a 15-day feed restriction molt. The lower rate observed in flock 2 may be explained by the shorter timespan after molting, the stress level during molt that is indicated by the high percentage of comb lesions and damaged plumage at the cloaca/belly region, the winter season and the condition of the hens. Logger data recorded temperatures below 10°C inside the mobile coop. Given that molting in the two commercial cohorts occurred in different seasons, the environmental and photoperiodic context warrants explicit consideration when interpreting the flock-specific responses. Seasonal cues, including ambient temperature, humidity and photoperiod (daylength), are known to modulate molt dynamics and post-molt recovery. In our commercial field cohorts, logger data documented distinct climatic contexts, with flock 1 experiencing higher mean temperatures (18.9 °C; range 5.0–35.3 °C) and flock 2 lower mean temperatures (13.6 °C; range 0.8–28.7 °C), including periods below 10 °C during molting, alongside similarly high relative humidity (∼78–81%; up to 100%). In addition, molting in flock 1 took place during summer under a markedly longer photoperiod, whereas flock 2 molted in autumn/winter with shorter daylength. Together, these seasonal differences may have contributed to variation in molt expression and recovery between cohorts; however, given the on-farm field design, they should be interpreted as real-world moderators rather than causal drivers. From a practical perspective, aligning molt induction with natural autumnal cues (declining photoperiod and lower ambient temperatures) may facilitate molt success, yet producers must balance this biological advantage against market demand (e.g., egg supply for the Christmas period) and the economic need to bridge the summer production gap, which can make summer molting strategically preferable despite less supportive natural cues. During molting, the hens in flock 2 appeared very weak, which led us to forgo the assessment of health indicators. Each hen was individually removed from the barn, weighed, radiographed, and then returned to the coop. Flock 1 showed in general a very low laying performance during the whole project time. Dual-purpose breeds typically exhibit laying performance ranging from approximately 54% to 73% under various management systems (Lambertz et al., 2018; Baldinger and Bussemas, 2021). However, post-molt laying performance data for dual-purpose breeds are scarce. It is known that molting induces a significant reduction in laying rates, with 74-78% reported in commercial layer strains (Biggs et al., 2003; Wu et al., 2007). The observed decrease in laying performance after molt in dual-purpose breeds aligns with these findings, indicating that reduced post-molt productivity could be a common physiological response. Yet, the lack of data underlines the need for further targeted research on the post-molt performance of dual-purpose hens. The weight gain after molting in both flocks could be interpreted as a sign of good recovering from the last laying cycle and molting. The weight gain observed in both flocks after molting may indicate successful recovery from the previous laying cycle and molt and is somehow in contrast to the low laying performance post molt. The weight gain is in line with the improvement of all health indicators in flock 1. In our study, the prevalence of injuries at the comb increased markedly after molting in flock 2 where injuries rose from 42.6% before molting to 78.5% after molting. This sharp increase contrasts with some literature reporting either stable or decreased comb injuries post-molt, like we observed in flock 1, which is often attributed to reduced aggression during the molt period (McCowan et al., 2006; Regmi et al., 2018). A likely explanation for the elevated comb injuries in our flock 2 lies in the strict molting management applied. The complete removal of feed (except for crushed summer barley) and immediate closure of free-range access on a single day likely induced increased stress and frustration among hens (Glatz and Tilbrook, 2021, Jensen et al., 2016). Similar findings were reported by Rodenburg et al. (2008), who linked sudden environmental restrictions and feed changes to increased aggressive interactions and injurious pecking. Furthermore, during our farm visits, it was observed that the feed chain was often completely empty during molting, which may have exacerbated competition and aggressive behaviors. This aligns with Zepp et al. (2018); Konkol et al. (2020) and Webster (2000), who emphasized that environmental enrichment and consistent feeding schedules can mitigate aggression and comb injuries in laying hens. The absence of such enrichment and feed scarcity during the molting period likely contributed to the high prevalence of comb injuries observed. Regarding other integument indicators such as feather loss and injuries on the back, cloaca, and footpads, our results showed moderate prevalence rates, with no significant changes during or after molting in most cases. In flock 1, the tendency for improvement across all integument health indicators post-molt further suggests a positive physiological development, consistent with findings by LaBrash and Schneider (2005) and Wang et al. (2023), who reported improved skin and feather condition associated with recovery phases after molting. Overall, the increased comb injuries post-molt in our study highlights the critical impact of molting management on hen welfare. Our findings suggest that abrupt feed restriction combined with immediate closure of outdoor access can intensify aggressive behaviors and stress, resulting in more frequent comb injuries. Therefore, tailored management strategies that ensure gradual feed reduction and maintain some level of environmental enrichment may be essential to reduce aggression and improve welfare during molting. Keel bone health was expected to improve during molt; however, our results clearly show an increase in fracture prevalence. This may be attributed to insufficient bone nutrition or elevated stress levels, which would be consistent with the observed increase in comb lesions in flock 2. We hypothesized that bone mineral density (BMD) would improve under optimal molting conditions, particularly with the provision of adequate calcium sources and sufficient daylight exposure. However, contrary to our expectations, no significant changes in BMD were observed in flock 1. This may be explained by the high heterogeneity within the flock and the relatively stable laying performance throughout the molting period. Bone density values in this group ranged from 5.2 to 6.1 mm aluminum equivalent (mm AlEq), reflecting a relatively high level of bone mineralization, in general. These values are notably higher than those reported by Eusemann et al. (2020), who found keel bone BMD levels between 1.0 and 4.0 mm AlEq, suggesting potentially better skeletal health in our study population or potential methodological differences in measurement. In flock 2, a significant increase in BMD was observed after molting, rising from 4.0 mm AlEq during molt to 5.7 mm AlEq post-molt. This result supports the potential for skeletal recovery under certain molting conditions. Nonetheless, our initial hypothesis—that BMD would improve during or immediately after molting – was only partially supported. In contrast to our findings, Mazzuco and Hester (2005) observed a significant reduction in tibial BMD during molting in White Leghorns, with full recovery requiring up to 128 days. These discrepancies may result from differences in the skeletal site assessed (keel vs. tibia), molting protocols, or genetic background of the birds used. Moreover, it remains unclear whether increased duodenal calcium uptake, as demonstrated by Al-Batshan et al. (1994) immediately post-molt, is directed primarily toward eggshell formation or also contributes significantly to bone remineralization. In our study, flock 2 showed an improvement in eggshell quality following molting, possibly reflecting this enhanced calcium utilization. If, however, reproductive demands consistently take precedence over skeletal integrity, enhanced dietary calcium alone may not suffice to ensure long-term bone health. In that case, genetic selection for hens that allocate a greater proportion of calcium and metabolic resources to skeletal maintenance would be a more sustainable strategy for improving bone stability in laying hens. The small amount of feed samples limits the validity of these results. It cannot be ruled out that a sample has become separated before analyses or that samples happened to contain a particularly large amount of a single nutrient. And since feed intake was not determined, it is not possible to determine the nutrient intake of the animals. Nevertheless, both flocks revealed relevant departures from recommended nutrient profiles, especially regarding the calcium-to-phosphorus (Ca:P) ratio, which is considered a key factor for bone health in laying hens (Bryden et al., 2021). In the samples of flock 1, calcium levels in the early molting ration were intentionally high (above 100 g/kg dry matter) and decreased appropriately during the post-molt phase, aligning with standard molting protocols. However, phosphorus concentrations remained elevated (up to 12.6 g/kg), likely exceeding the levels of digestible phosphorus required. As a result, the Ca:P ratio in this flock was likely lower than the optimal 2:1 to 3.5:1 range recommended to support bone mineralization (Bryden et al., 2021). An imbalance was also observed in flock 2. Although crude protein and most amino acid levels were above recommendations, calcium levels were suboptimal (25.5 and 29.5 g/kg TS) in both sampled rations before and after molting, while phosphorus content remained excessive (4.8 and 5.4 g/kg TS) in both. This combination further reduces the Ca:P ratio and may limit calcium absorption and its deposition in bone tissue, even when calcium intake is quantitatively adequate. Such imbalances can lead to impaired bone development, as excess phosphorus increases intestinal pH and interferes with calcium solubility and uptake (Bryden et al., 2007). The persistently low Ca:P ratios observed in both flocks could therefore have contributed to the lack of significant improvement in bone mineral density (BMD) after molting, particularly in flock 1. Moreover, despite an observed increase in BMD in flock 2 after molting, the overall imbalance between calcium and phosphorus may have limited the magnitude of skeletal recovery. These findings underscore the importance of not only ensuring sufficient calcium supply but also avoiding excessive phosphorus content to support bone health and structural integrity during and after the molting phase. However, because this was a commercial on-farm field study, feed data can only be interpreted as contextual information on ration composition. Feed analyses provide important background on diet formulation in commercial systems, but individual feed intake could not be quantified under free-range conditions; therefore, nutrient-related interpretations (e.g., calcium levels) must be regarded as associative rather than causal. Future work combining commercial field conditions with precision-feeding or intake monitoring would allow stronger inference on individual mineral uptake. This study had further methodological limitations. Our custom-made x-ray frame proved useful, as hens tolerated suspension well, spreading their wings to allow clear imaging of the keel bone. However, the adjustable design – intended to accommodate hens of varying size and weight – introduced slight variability in positioning. To ensure consistent image angles, fixed alignment markers are strongly recommended. The radiographic equipment should also be shielded from dust and dirt to facilitate cleaning and disinfection; technical recommendations are available from the corresponding author. Individual identification via numbered leg bands was essential to track changes over time. We initially used spiral bands, which detached from some birds, and later replaced them with snap-on rings. Depending on the housing system, not all hens may be captured during each visit, reducing sample size further. Hence, a sufficiently large sample should be planned in advance to account for this. These limitations highlight the difficulty of collecting reliable data in commercial flocks without a clearly defined and coordinated molting protocol. In future trials, molting plans should be co-developed and documented together with the farmer. It is also crucial to collect management and performance data in real time, as retrospective information from farmers often lacks accuracy. Farmers should be provided with data collection record sheets before project onset. The influence of season and climate on molting dynamics and hen condition warrants further investigation. While flock 1 appeared healthy and balanced throughout, flock 2 showed signs of poor general condition, which hindered data collection at certain time points. The reliability of visual assessment methods for molting status – such as feather cover on the neck and underwings, presence of new feather shafts, cloacal condition, and pubic bone spacing – should be validated against objective techniques like ultrasonography. Finally, although flock 2 exhibited higher post-molt laying performance, flock 1 showed generally very low egg production throughout the observation period, with no substantial differences between sampling time points. Nevertheless, the more gradual and welfare-conscious induction of molting in flock 1 contributed to a noticeably calmer environment for hens, farmers, and researchers alike.

## Conclusion

Further on-field investigations should plan sample sizes that account for potentially higher mortality than usual and consider using click-leg rings for individual marking. Additionally, it is crucial to fix alignment markers at the X-ray frame to ensure a consistent X‑ray angle. In commercial practice, the effects of induced molting on laying hen health, welfare, and performance appear to depend on flock condition and the specific molting protocol applied. Improvements in egg quality traits following molt suggest a recovery of reproductive tract function and physiological performance. However, the potential of induced molting to enhance bone health remains uncertain. Even in cases where bone mineral density remains stable or increases, the number and severity of keel bone fractures may still rise. These findings underscore the need for refined molting strategies that balance performance recovery with skeletal health and animal welfare.

## Funding

This research was funded by the central research fund ZFF of the University of Kassel under grant number 3109.

## CRediT authorship contribution statement

**Julia Gickel:** Writing – review & editing, Writing – original draft, Visualization, Formal analysis. **Frieder Broszat:** Investigation, Data curation. **Louisa Salzmann:** Visualization, Formal analysis, Data curation. **Nicole Kemper:** Writing – review & editing. **Lisa Jung:** Writing – original draft, Supervision, Project administration, Methodology, Investigation, Funding acquisition, Formal analysis, Data curation, Conceptualization.

## Disclosures

The authors declare that they have no known competing financial interests or personal relationships that could have appeared to influence the work reported in this paper.

## Appendix Supplementary materials

Image, image 1

Image, image 2

Image, application 3

Image, image 4
