# Supplementary figures and images for: Extending laying hens’ productive lifespan by molting: a pilot on-farm study and test of assessment methods with focus on functional traits and keel bone health
Source: Poult Sci. 2025 Dec 20;105(2):106323. doi: 10.1016/j.psj.2025.106323 (PMC12799921; doi:10.1016/j.psj.2025.106323)

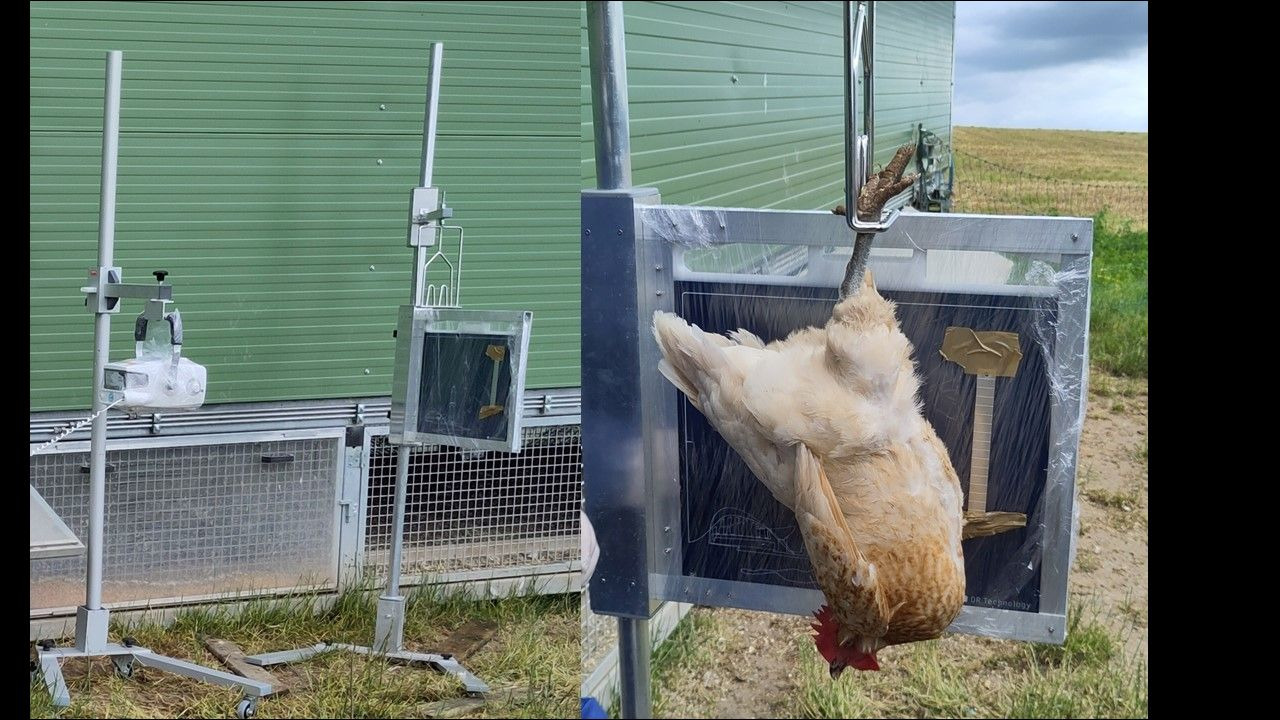

Supplement: Supplementary file 1 [file mmc1.jpg]

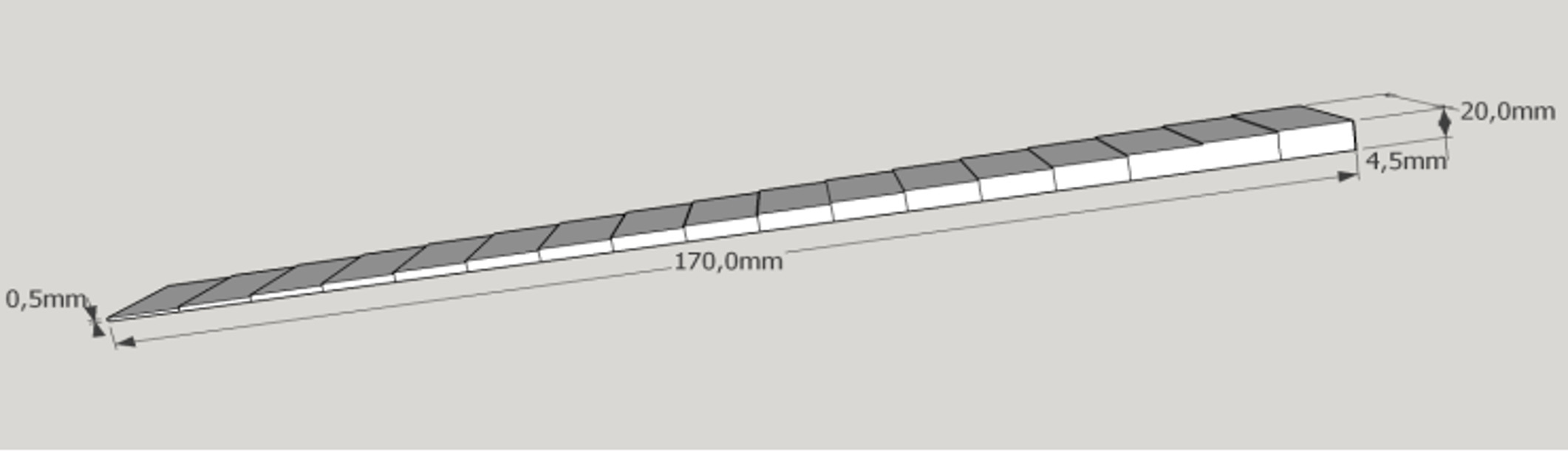

Supplement: Supplementary file 2 [file mmc2.jpg]

## Slide 1
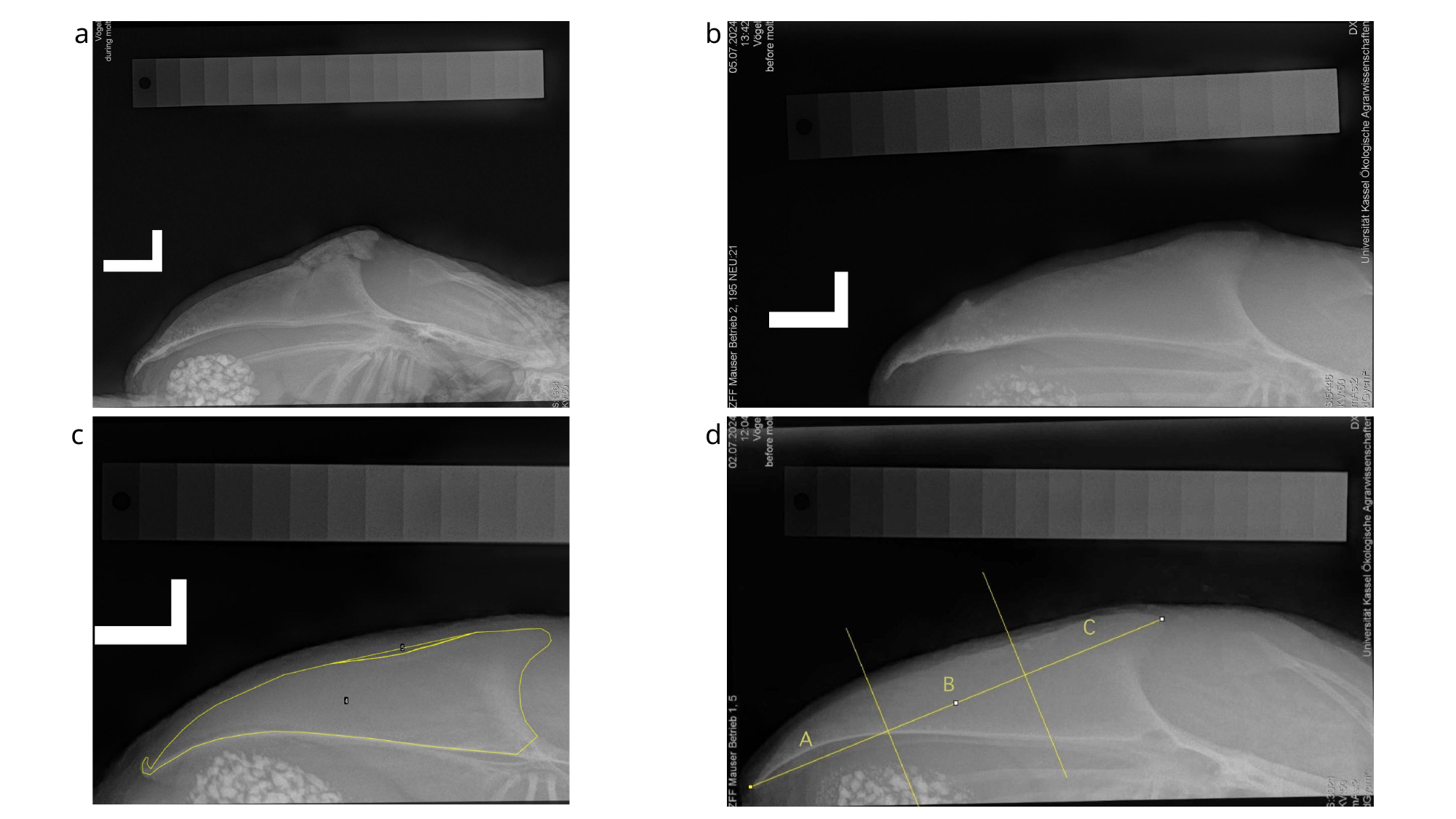

b
a
c
d

Supplement: Supplementary file 3 [file mmc3.pptx]

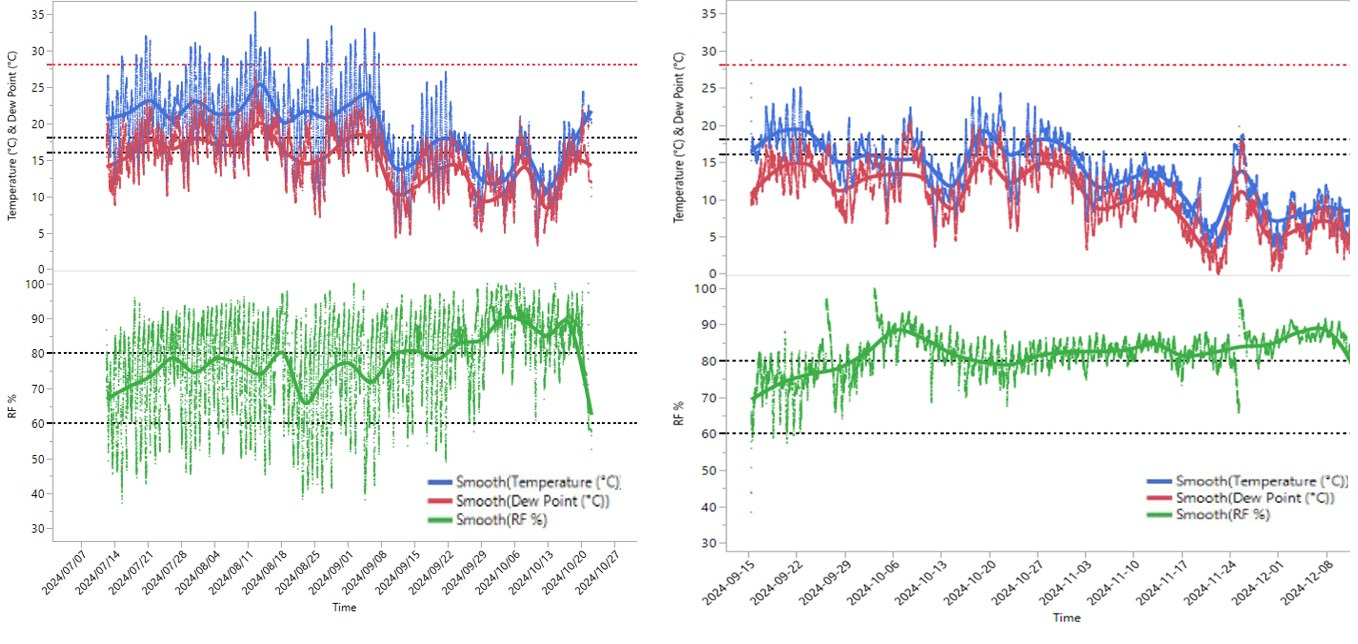

Supplement: Supplementary file 4 [file mmc4.jpg]
